# EHMTI-0287. Peptides involved in sleep and appetite homeostatic regulation and its effects in the modulation of trigeminovascular nociceptive activation

**DOI:** 10.1186/1129-2377-15-S1-F18

**Published:** 2014-09-18

**Authors:** M Martins Oliveira, S Akerman, J Hoffmann, P Goadsby

**Affiliations:** 1 Sandler Neuroscience Center, University of California San Francisco, San Francisco, USA

## Background

Disturbances in appetite and sleep contribute to triggering in migraineurs. Neuropeptide Y (NPY), leptin and insulin are involved in hypothalamic appetite and sleep regulation, and pain modulation. The orexinergic system, known to modulate trigeminocervical complex (TCC) nociceptive activation, is reciprocally influenced by NPY, leptin and insulin. Importantly, hypothalamic activation is involved in migraine pathophysiology.

## Aim

To determine the effect of homeostatic peptides on TCC neuronal activity in response to middle meningeal artery (MMA) dural electrical stimulation.

## Methods

Sprague-Dawley rats were anesthetized, the parietal bone was removed over the MMA for dura mater electrical stimulation, and TCC neurons were recorded. Rats received either NPY human, NPY Y1 receptor agonist, rat recombinant leptin or human insulin, or sterile water as control. Glycemic levels were monitored when insulin was administered. Studies approved by the IACUC.

## Results

Dural-evoked neuronal firing in the TCC was significantly reduced by NPY human (30 μg/kg, p < 0.05, max inhibition 18%), NPY Y1 receptor agonist (30 μg/kg, p < 0.005, max inhibition 22%), leptin (1 mg/kg, p < 0.02, max inhibition 13%) and human insulin (10 U/kg, p = 0.000, max inhibition 17%).

## Conclusion

These studies demonstrate that NPY, an NPY Y1 agonist, leptin and insulin cause perturbations in stimulus-evoked trigeminal activity. Hypothalamic bidirectional projections to the TCC may regulate trigeminovascular nociceptive traffic through NPY, leptin and insulin signaling. These data demonstrate a link between the potential origin of disturbed feeding and sleep regulation in migraine and the involvement of homeostatic peptides in trigeminovascular nociceptive traffic modulation.

No conflict of interest.

